# Adherence to COVID-19 vaccination during the pandemic and fake news: Correspondence

**DOI:** 10.1590/0034-7167.202477Suppl101c

**Published:** 2025-01-10

**Authors:** Hinpetch Daungsupawong, Viroj Wiwanitkit

**Affiliations:** I Private Academic and Editorial Consultant. Phonhong, Laos; II Chandigarh University Gharuan, University Centre for Research & Development Department of Pharmaceutical Sciences. Mohali, Punjab, India

Dear Dr Dulce Aparecida Barbosa

Editor in Chief of the Revista Brasileira de Enfermagem

We would like to share ideas on the publication “Adherence to COVID-19 vaccination during the pandemic: the influence of fake news”^(1)^.This study, carried out in Campo Grande, Mississippi, looked at how nursing professionals saw the population’s reaction to vaccine reluctance. According to the findings, experts saw a rise in vaccination reluctance in the public, which they blamed on the impact of fake news and denialist activities. It was discovered that the public’s trust in vaccinations and the medical personnel who deliver them is adversely impacted by these circumstances. Twenty nursing professionals participated in semi-structured interviews for the study, and the data was analyzed using thematic content analysis.

The study’s tiny sample size of only twenty nursing professionals is one possible weakness. This small sample might not fully represent the views of all nursing professionals in Campo Grande, MS, or represent the entire spectrum of viewpoints about vaccine hesitancy. Furthermore, the study’s conclusions can include biases and inconsistencies because it only used the nursing professionals’ self-report data. Expanding the sample size and utilizing various data collection techniques should be part of future study in this field to guarantee the accuracy and consistency of the results.

The study’s exclusive focus on the opinions of nursing professionals, without taking into account those of other healthcare providers or community members, is another drawback. Incorporating a variety of viewpoints may yield a more thorough comprehension of the variables influencing vaccination reluctance within the populace. Furthermore, the study did not examine any possible tactics or solutions to deal with vaccine reluctance, which can be a useful topic for further investigation. Researching efficient strategies to dispel myths and boost vaccine acceptability could contribute to the development of public health programs and regulations meant to raise immunization rates.

It is agreeable that it is need to properly manage the fake news and it requires attention from all parties. Regarding vaccination, it sometimes very complex. Due to the advent of social media technology, it is difficult to manage. Several anti-COVID-19 blogposts exist and circulate. In a more serious situation, a blogpost owner might disguise his/her own conflict of interest and write/pay for publication of an article in a standard journal with hidden agenda to support his/her own website business and the published work might be further references with risk to the general readers. The qualification of the author of an article is the important thing to be focused^(2)^.

Regarding future directions, it would be advantageous for researchers to carry out a follow-up study in order to investigate the effects of denialist actions and fake news on vaccination hesitancy in the general public. This can entail looking into the precise sources of false information and how they affect how the general public views vaccines. Future studies could also look into how well various communication tactics work to overcome vaccine skepticism and encourage vaccine adoption. Healthcare providers and legislators can endeavor to raise vaccination rates and boost public confidence in vaccinations by identifying successful treatments and tactics.
