# Supplementary material for: Adherence to COVID-19 vaccination during the pandemic and fake news: Correspondence
Source: Rev Bras Enferm. 2025 Jan 10;77(Suppl 1):e202477Suppl101c. doi: 10.1590/0034-7167.202477Suppl101c (PMC11726812; doi:10.1590/0034-7167.202477Suppl101c)
Supplement: Related texts

# Reply: AUTHORS' RESPONSE

**Authors:** Elen Ferraz Teston, Sonia Silva Marcon, Luana Cristina Roberto Borges, Gabrielly Segatto Brito

**Affiliations:** I Universidade Federal de Mato Grosso do Sul. Campo Grande, Mato Grosso do Sul, Brazil; II Universidade Estadual de Maringá. Maringá, Paraná, Brazil

Dear Professor Doctor Dulce Aparecida Barbosa

Editor-in-Chief of the Revista Brasileira de Enfermagem

In response to the letter to the editor regarding our article titled *Adherence to COVID-19 Vaccination During the Pandemic and Fake News*, published in volume 77, number 1, we present the following considerations:

1. Small sample size: The small sample size, consisting of only twenty nursing professionals, may be seen as a limitation, as it does not represent the opinion of all nursing professionals in Campo Grande, MS, regarding vaccine hesitancy.
  - We remind you that qualitative studies are not intended to make generalizations, and for this reason, the statements made throughout the article were always presented in a relativistic manner. Furthermore, the methodology explicitly mentioned that the sample consisted of nursing professionals involved in immunization across nine urban health units, which make up one of the seven Health Districts in the municipality. Qualitative studies generate hypotheses that can be confirmed or refuted through studies using other designs and robust statistical methods.
2. The study’s conclusions may include biases and inconsistencies because it used only self-reported data from nursing professionals. Expanding the sample size and using different data collection techniques should be part of future studies in this area to ensure the accuracy and inconsistency of the results.
  - It is worth noting that obtaining information through self-reports (interviews) is the most widely used data collection technique in qualitative studies. Furthermore, the suggestions presented may be considered in future quantitative studies, since qualitative studies do not aim to guarantee the accuracy of the results. Thus, the difference in qualitative research lies in the singularities and meanings attributed to experiences and the appreciation of the sociocultural dimensions of certain events^(1)^.
3. The study’s exclusive focus on the opinions of nursing professionals, without taking into account those of other health care providers in the community. This is another disadvantage.
  - We agree that incorporating a variety of points of view could produce a more in-depth understanding of the factors that influence the population’s reluctance to vaccination. For this reason, the participation of only nursing professionals in the study was pointed out as a possible limitation of the study, as we believe that the participation of other professionals and even users of the service could contribute to broadening the perspective regarding adherence to vaccination. Despite the limitation presented, the results found corroborate (from a qualitative perspective) other studies, worldwide, in relation to the drop in vaccination rates and vaccine hesitancy(2-4).
  - Furthermore, we believe that in order to broaden the understanding of the phenomenon, in addition to incorporating professionals with different backgrounds, it would be more appropriate to conduct a study that adopted another methodological framework, for example, Grounded Theory, rather than a descriptive study like the one carried out here.
  - The objective of the study was to understand how fake news influenced adherence to immunization against COVID-19, from the perspective of health professionals. Therefore, exploring possible tactics or solutions to deal with reluctance to vaccinate is an object to be explored in another study with a specific purpose. - We agree that the investigation of strategies to dispel myths and increase the acceptability of vaccines can contribute to the development of public health programs and regulations aimed at increasing immunization rates. However, we remember that the present study originated from a professional master’s dissertation presented to a Postgraduate Program in Family Health and that students at this level of education are able to develop studies that do not require much time for data collection. It is also important to highlight that in order to obtain the master’s degree, the main author defended her dissertation before an evaluation committee composed of the advisor and two other professors (one from an external institution) and that the evaluation of the final product was based on ethical principles and good practices in the development of scientific research.

## References

[1] Minayo MCS. (2017). Amostragem e saturação em pesquisa qualitativa: consensos e controvérsias. Revista Pesquisa Qualitativa.

[2] Procianoy GS, Rossini F, Lied AF. (2022). Impact of the COVID-19 pandemic on the vaccination of children 12 months of age and under: an ecological study. Cien Saude Colet.

[3] Silveira MF, Tonial CT, Goretti K. (2021). Maranhão A. Missed childhood immunizations during the COVID-19 pandemic in Brazil: analyses of routine statistics and of a national household survey. Vaccine.

[4] Santos VS, Vieira SCF, Barreto IDC. (2021). Effects of the COVID-19 pandemic on routine pediatric vaccination in Brazil. Expert Rev Vaccines.
